# Association Between Pregnancy Hope and Premenstrual Dysphoric Disorder Among Female Workers: A Cross‐Sectional Online Survey in Japan

**DOI:** 10.1002/npr2.70106

**Published:** 2026-03-06

**Authors:** Yuka Ito, Natsu Sasaki, Yoshiaki Kanamori, Rikako Tsuji, Mako Iida, Kazuhiro Watanabe, Miho Egawa, Daisuke Nishi

**Affiliations:** ^1^ Department of Mental Health, Graduate School of Medicine The University of Tokyo Bunkyo‐ku Tokyo Japan; ^2^ Department of Psychiatric Nursing, Graduate School of Medicine The University of Tokyo Bunkyo‐ku Tokyo Japan; ^3^ Department of Public Health, School of Medicine Kitasato University Sagamihara‐shi Kanagawa Japan; ^4^ Department of Gynecology and Obstetrics, Graduate School of Medicine Kyoto University Kyoto Japan

**Keywords:** pre‐conception, pregnancy hope, premenstrual dysphoric disorder, women's health

## Abstract

**Objectives:**

This cross‐sectional study examined the association between pregnancy hope and premenstrual dysphoric disorder (PMDD) outcomes among full‐time working women.

**Methods:**

We analyzed an October 2023 survey of nulligravid women aged 20–44 years (*N* = 1947). PMDD diagnosis and symptom severity were assessed using a DSM‐IV‐based scale. Logistic and linear regression examined associations with pregnancy hope and tested interaction by marital status.

**Results:**

PMDD prevalence was 5.4% with pregnancy hope and 4.8% without. Pregnancy hope was not associated with PMDD diagnosis (crude OR = 1.13, 95% CI: 0.71–1.80) and remained null after adjustment. Pregnancy hope was weakly associated with higher symptom scores (crude standardized *β* = 0.059, *p* = 0.009), persisting after adjustment. Interaction by marital status was not significant.

**Conclusion:**

Among full‐time workers, pregnancy hope was unrelated to PMDD diagnosis but was weakly associated with subthreshold symptom severity. Longitudinal studies that comprehensively assess multidimensional pregnancy intention, perceptions of menstruation, and work‐related constraints are needed to clarify causal directionality and mechanisms.

## Abstract 2

In a survey of 1947 female workers, pregnancy hope was not associated with a PMDD diagnosis but showed a weak association with greater severity of subthreshold symptoms. To clarify causal directionality and underlying mechanisms, longitudinal studies are needed that comprehensively assess multidimensional pregnancy intentions, perceptions of menstruation, and work‐related constraints.

## Graphical abstract

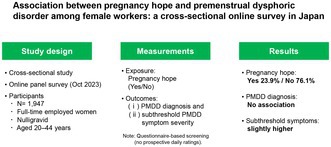

## 1 Introduction

Menstruation‐related symptoms significantly impact women's physical and mental well‐being, with up to 90% experiencing discomfort during premenstrual and menstrual periods [1, 2]. Premenstrual dysphoric disorder (PMDD) represents the most severe form, characterized by affective symptoms with clinically significant distress or interference [3, 4]. The 12‐month prevalence of PMDD ranges from 1.8% to 5.8% [4] and is associated with increased suicide risk [5] and substantial productivity loss [6]. Moreover, even subthreshold symptoms can impair daily functioning [7]. As pharmacotherapy alone may be insufficient in some cases and some patients prefer non‐drug therapy due to side effects or pregnancy hope [8, 9], it is essential to explore further psychological factors in PMDD.

The onset of menstruation signifies that conception did not occur during that cycle. For women actively trying to conceive, menstruation represents an event where the actual self diverges from the ideal self or the ought self, according to Higgins' self‐discrepancy theory [10]. This discrepancy may reinforce negative attitudes toward menstruation, which are associated with more severe premenstrual symptoms [11, 12]. In addition, women hoping to become pregnant may also more actively anticipate menstruation as a marker of conception outcome, which may in turn heighten negative affect [13]. Furthermore, some women who are not currently trying to conceive but nevertheless have a strong wish to become pregnant may experience menstruation as a recurrent reminder of reproductive capacity and fertility‐related concerns [14]. Anxiety and stress have also been reported to exacerbate menstruation‐related symptoms [15], and such psychological factors may in turn contribute to more severe PMDD symptoms among women who experience concerns about their fertility. In Japan, only about half of full‐time working women reportedly conceived when they wished to [16], suggesting that conflicts between pregnancy desire and reality may be common in working populations.

However, only a few studies have examined related constructs. Two older studies compared women undergoing infertility treatment (not specific to workers) with controls and found no clear differences in menstruation‐related symptoms [17, 18], but these studies were conducted over 30 years ago and did not adjust for covariates. Marital status may influence both pregnancy hope and emotional responses to menstruation, for example through actual attempts to conceive and partnership‐related factors, but its role in this association remains unclear.

Thus, this study aimed to investigate the association between pregnancy hope and PMDD‐related outcomes among full‐time female workers in Japan. We hypothesized that pregnancy hope would increase PMDD diagnosis and symptom severity. We also conducted an exploratory assessment of heterogeneity by marital status.

## 2 Materials and Methods

### 2.1 Study Design and Participants

This cross‐sectional study used data from an October 2023 survey of a Japanese online panel (Cross Marketing Inc., *N* = 5.6 million). The survey recruited women aged 20–44 years who were nulligravid, employed full‐time, and had no oral contraceptive use in the past year. Recruitment closed when 2000 women completed the questionnaire. The protocol was approved by the Research Ethics Committee of the Graduate School of Medicine and Faculty of Medicine at the University of Tokyo (No. 2023058NI‐(1)). We followed the Strengthening the Reporting of Observational Studies in Epidemiology (STROBE) [19].

### 2.2 Measurements

#### 2.2.1 Pregnancy Hope

Participants were asked, “Are you hoping to become pregnant now?” (yes/no), a question used previously [20].

#### 2.2.2 Premenstrual Dysphoric Disorder

PMDD symptoms were assessed using a validated Japanese PMDD scale based on DSM‐IV criteria [21, 22]. Section A consists of 12 symptom items rated from 1 (none) to 4 (severe), and Section B includes five items assessing functional impairment in work, social activities, and relationships. Participants were classified as having PMDD if they reported at least one mood symptom in Section A rated as severe, at least four Section A items rated as moderate or severe, and at least one Section B item rated as severe. We also used the Section A total score as a continuous indicator of PMDD symptom severity [23].

#### 2.2.3 Demographic Variables

Based on previous literature [24], we assessed age, educational attainment (high school or lower vs. university or higher), marital status (unmarried/married), household income, smoking status (non‐smoker/smoker), body mass index (BMI), current psychiatric illness (yes/no), and history of gynecological illness other than premenstrual syndrome (yes/no).

### 2.3 Statistical Analysis

Participant characteristics were described using means (SD) or frequencies. PMDD diagnosis (binary outcome) was analyzed using logistic regression, and PMDD symptom scores (continuous outcome) were analyzed using multivariable linear regression. To clearly compare heterogeneity by marital status between unmarried and married groups, divorced or bereaved participants were excluded from the primary analyses and included in sensitivity analyses. We fitted three models: Model 1 adjusted only for age, and Model 2 additionally adjusted for educational attainment, marital status, household income, smoking status, BMI, and history of gynecological illness other than premenstrual syndrome. Model 3 added current psychiatric illness to Model 2 as a sensitivity analysis, as it may lie on the causal pathway. We tested pregnancy hope × marital status interaction terms in Model 2. Statistical significance was defined as a two‐sided *p* < 0.05. Data were analyzed using SPSS version 29.0 (Armonk, NY: IBM Corp).

## 3 Results

Of the 2000 women who completed the survey, 1947 were included in the main analyses after excluding divorced or bereaved participants. The mean age was 33.4 years (SD = 6.3), with the majority being unmarried (82.0%). Pregnancy hope was reported by 23.9% of participants. Women hoping to become pregnant were slightly younger and more likely to be married and to report a history of gynecological illness (Table 1).

**TABLE 1:** Demographic characteristics by pregnancy hope (*N* = 1947).

	Pregnancy hope	
	No (*N* = 1482, 76.1%)	Yes (*N* = 465, 23.9%)
	*n* (%)	
Age, mean (SD)	33.80 (6.65)	33.00 (5.52)
20–29	462 (31.2)	138 (29.7)
30–39	631 (42.6)	260 (55.9)
40–44	389 (26.2)	67 (14.4)
Marital status		
Unmarried	1281 (86.4)	316 (68.0)
Married (including common law marriage)	201 (13.6)	149 (32.0)
Education status		
Junior high/high school	214 (14.4)	58 (12.5)
Technology/vocational/junior college	372 (25.1)	104 (22.4)
University	832 (56.1)	285 (61.3)
Graduate school	64 (4.3)	18 (3.9)
Household income (JPY)		
< 200	80 (5.4)	19 (4.1)
200 to < 400	398 (26.9)	99 (21.3)
400 to < 600	376 (25.4)	123 (26.5)
600 to < 800	257 (17.3)	94 (20.2)
800 to < 1000	173 (11.7)	63 (13.5)
≥ 1000	198 (13.4)	67 (14.4)
Smoking		
Non‐smoker	1369 (92.4)	443 (93.1)
Smoker	113 (7.6)	32 (6.9)
Current psychiatric illness		
No	1376 (92.8)	438 (94.2)
Yes	106 (7.2)	27 (5.8)
Gynecological illness history (except for PMS)		
No	1276 (86.1)	376 (80.9)
Yes	206 (13.9)	89 (19.1)
BMI, mean (SD)	20.56 (3.91)	20.40 (3.30)

Overall, PMDD prevalence was 4.9%, and was 5.4% among women with pregnancy hope and 4.8% among those without. In the crude analysis, pregnancy hope was not significantly associated with PMDD diagnosis (OR = 1.13, 95% CI: 0.71–1.80, *p* = 0.611). This null association persisted in both Model 1 and Model 2 (Table 2).

**TABLE 2:** Association between pregnancy hope and PMDD outcomes (*N* = 1947).

	Crude						Adjusted					
	Pregnancy hope				OR [95% CI]	*p*	Model 1^a^		Model 2^b^		Model 3^c^	
	No (*n* = 1482, 76.1)		Yes (*n* = 465, 23.9%)									
	*n* (%)		*n* (%)				aOR [95% CI]	*p*	aOR [95% CI]	*p*	aOR [95% CI]	*p*
PMDD diagnosis	71 (4.8)		25 (5.4)		1.13 [0.71–1.80]	0.611	1.10 [0.70–1.76]	0.680	1.11 [0.68–1.80]	0.677	1.14 [0.70–1.84]	0.610

	Mean	SD	Mean	SD	Standardized *β* ^d^	*p*	Standardized *β* ^d^	*p*	Standardized *β* ^d^	*p*	Standardized *β* ^d^	*p*
PMDD section A	22.28	9.19	23.54	9.11	0.059	0.009*	0.053	0.019*	0.054	0.019*	0.057	0.013*

^a^ Adjusted for age.

^b^ Adjusted for age, education status, marital status, household income, smoking, body mass index, and gynecological illness history except for premenstrual syndrome.

^c^ Adjusted for age, education status, marital status, household income, smoking, body mass index, gynecological illness history except for premenstrual syndrome, and current psychiatric illness.

^d^ Standardized *β* was calculated by using regression analysis.

* *p* < 0.05.

On the other hand, PMDD symptom scores were higher among women hoping for pregnancy than among those not hoping for pregnancy (mean 23.54 vs. 22.28). Pregnancy hope was associated with higher scores in the crude analysis (standardized *β* = 0.059, *p* = 0.009), and this association persisted in the age‐adjusted model (Model 1: *β* = 0.053, *p* = 0.019) and primary adjusted model (Model 2: *β* = 0.054, *p* = 0.019) (Table 2). We also tested for interaction by marital status; interaction terms were not significant for PMDD diagnosis (*p* = 0.896) or symptom scores (*p* = 0.162).

Results were similar after adjustment for current psychiatric illness (Model 3) and when divorced/bereaved women were included (Table 2; Table S1).

## 4 Discussion

We examined the association between pregnancy hope and PMDD outcomes among full‐time workers. PMDD prevalence (4.9%) was within prior questionnaire‐based estimates for Japanese working women (e.g., 3.5%–13.7%) [25, 26]. Pregnancy hope was not associated with PMDD diagnosis but was associated with more severe subthreshold PMDD symptoms, partially supporting our hypothesis.

PMDD diagnostic criteria are stringent, requiring ≥ 5 symptoms including mood symptoms and functional impairment; yet women below this threshold may still experience meaningful distress, which a dichotomous PMDD/non‐PMDD classification can miss [3, 27, 28]. That said, the observed difference was small (crude: standardized *β* = 0.059; Cohen's *d* = 0.14), and the minimal clinically important difference for this scale is unknown; thus, further work is needed to establish clinical significance, and our findings should be interpreted cautiously.

Several hypotheses may be consistent with the association between pregnancy hope and subthreshold symptoms. Lustyk et al. [13] reported that individuals who more actively anticipate menstruation based on physical and psychological changes experience heightened negative affect. Especially among women actively trying to conceive, menstruation may be interpreted as a sign of not having conceived and may be associated with more negative attitudes toward menstruation and greater perceived premenstrual mood symptoms. Even among those who are not yet trying to conceive, menstruation may act as a recurrent reminder of future reproductive potential and the perceived time window for childbearing, potentially contributing to anxiety about future infertility and conflicts between pregnancy plans and career demands [14]. Such background stress may be associated with greater perceived severity of physical and emotional symptoms around menstruation.

At the same time, “pregnancy desire” was assessed using a single dichotomous item that could not capture key dimensions such as intention intensity or urgency, active trying‐to‐conceive, anticipated timing, or fertility concerns. The observed association may therefore reflect heterogeneous hopes and concerns tied to self‐perceived pregnancy desire; this exposure heterogeneity could have attenuated the association or produced effects driven by unidentifiable subgroups. Future studies should employ multidimensional measures of pregnancy intention (e.g., desire and timing) [29], and assess active trying‐to‐conceive status and fertility concerns.

The non‐significant interaction between pregnancy hope and marital status may suggest that this association is not explained solely by the presence of a spouse; however, the small number of married women and PMDD cases likely left the study underpowered to detect effect modification.

### 4.1 Limitations

This study has limitations. First, generalizability is limited because we excluded part‐time workers and housewives, and the opt‐in web survey may have overrepresented digitally engaged women (e.g., urban residents with higher education) despite nationwide recruitment [30]. Second, given the cross‐sectional design, directionality cannot be established and reverse causation is possible (e.g., severe premenstrual symptoms may shape pregnancy‐related attitudes). Third, PMDD was assessed using a DSM‐IV‐based questionnaire (not validated against DSM‐5).

Moreover, definitive PMDD diagnosis requires prospective daily ratings over ≥ 2 cycles; thus, findings represent questionnaire‐based screening results, not clinical diagnoses. Fourth, adjusting for current psychiatric disorders may have underestimated associations. Fifth, pregnancy desire was assessed by a single dichotomous item and may not capture key dimensions of pregnancy intentions (e.g., intensity/urgency and active trying‐to‐conceive), which may limit interpretation.

## 5 Conclusion

Among Japanese full‐time female workers, pregnancy hope was not associated with PMDD diagnosis but was weakly associated with severity of subthreshold symptoms. To clarify causal directionality and mechanisms, longitudinal studies are needed that comprehensively assess multidimensional pregnancy intention, perceptions of menstruation, and work‐related constraints.

## Author Contributions

N.S. supervised the study. All authors designed the study. Y.I. analyzed data and drafted the manuscript. All authors revised and approved the final manuscript.

## Funding

Grant‐in‐Aid for Scientific Research from the Japan Society for the Promotion of Science (JSPS) (23K16350). The funder had no role in the study.

## Ethics Statement

University of Tokyo Ethics Committee (no. 2023058NI‐(1)).

## Consent

Participant consent statement: obtained.

## Conflicts of Interest

Y.I. reports remuneration from Ikiiki Work Inc.; personal fees from Chiyoda Life Adjust Co. Ltd., Agonist Co. Ltd., iCARE Co. Ltd., and Manaby Co. Ltd. N.S. has received fees from Medilio Co. Ltd. D.N. has received fees from MD.net and honoraria from Takeda Pharmaceutical Co. Ltd. and Otsuka Medical Devices Co. Ltd. All are outside the submitted work. The remaining authors declare no conflicts of interest.

## Supporting information


**Table S1:** Association between pregnancy hope and PMDD outcomes including divorced/bereaved participants (sensitivity analysis; *N* = 2000).

## Data Availability

The data that support the findings of this study are available on request from the corresponding author. The data are not publicly available due to privacy or ethical restrictions.
