# Supplementary material for: Association Between Pregnancy Hope and Premenstrual Dysphoric Disorder Among Female Workers: A Cross‐Sectional Online Survey in Japan
Source: Neuropsychopharmacol Rep. 2026 Mar 6;46(1):e70106. doi: 10.1002/npr2.70106 (PMC12964171; doi:10.1002/npr2.70106)
Supplement: Supplementary file 1 — Table S1: Association between pregnancy hope and PMDD outcomes including divorced/bereaved participants (sensitivity analysis; N = 2000). [file NPR2-46-e70106-s001.docx]

Table S1. Association between pregnancy hope and PMDD outcomes, including divorced/bereaved participants (sensitivity analysis; N = 2,000)

|  | **Crude** | | | | | | **Adjusted** | | | |  |  |
| --- | --- | --- | --- | --- | --- | --- | --- | --- | --- | --- | --- | --- |
|  | **Pregnancy hope** | | | |  |  |  |  |  |  |  |  |
|  | **No (n=1522,76.1%)** | | **Yes (n=478, 23.9%)** | |  |  | **Model1^a^** | | **Model2^b^** | | **Model3^c^** | |
| **PMDD diagnosis** | **n (％)** | | **n (％)** | | **OR [95% CI]** | ***p*** | **aOR [95% CI]** | ***p*** | **aOR [95% CI]** | ***p*** | **aOR [95% CI]** | ***p*** |
|  | 74 (4.9) | | 27 (5.6) | | 1.17 [0.75-1.84] | 0.494 | 1.14 [0.73-1.80] | 0.567 | 1.14 [0.71-1.81] | 0.597 | 1.16 [0.72-1.86] | 0.539 |
| **PMDD section A** | **Mean** | **(SD)** | **Mean** | **(SD)** | **Standardized β^d^** | ***P*** | **Standardized β^d^** | ***p*** | **Standardized β^d^** | ***p*** | **Standardized β^d^** | ***p*** |
|  | 22.28 | 9.19 | 23.54 | 9.11 | 0.059 | 0.009* | 0.052 | 0.020* | 0.052 | 0.021* | 0.055 | 0.015* |

a Adjusted for age

b Adjusted for age, education status, marital status, household income, smoking, body mass index, and gynecological illness history except for premenstrual syndrome

c Adjusted for age, education status, marital status, household income, smoking, body mass index, gynecological illness history except for premenstrual syndrome, and current psychiatric illness

d Standardized β was calculated by using regression analysis

* p < 0.05
